# Nasopharyngeal swab for the diagnosis of SARS-CoV-2 (COVID-19) infection complicated by severe pneumococcal meningitis

**DOI:** 10.1016/j.idcr.2025.e02381

**Published:** 2025-09-27

**Authors:** Comlan Affo, Jeanne Tisseau des Escotais, Antoine Bosquet, Isabelle Mahé

**Affiliations:** a Service de Médecine Interne, Hôpital Louis Mourier, AP-HP, Colombes, France; b Université Paris Cité, Paris, France; c Innovative Therapies in Hemostasis, INSERM UMR_S1140, Paris, France; d INNOVTE-FCRIN, Paris, France

**Keywords:** Nasopharyngeal swab, COVID-19, Bacterial meningitis, Encephalocele

## Abstract

The nasopharyngeal swab is the reference test for diagnosing coronavirus disease 2019 (COVID-19) and other respiratory pathogens. Although this procedure appears to be risk free and was performed thousands of times daily during peaks of the COVID-19 pandemic, as well as during influenza and bronchiolitis outbreaks, it can be associated with minor to serious risks. These range from simple epistaxis to breaches of the meninges and skull stalks. Some breaches occur in the presence of anomalies such as congenital, traumatic or surgical encephaloceles. Neuro-meningeal breaches can be complicated by bacterial infections. We report the case of a 57-year-old woman diagnosed with *Streptococcus pneumoniae* meningitis following a nasopharyngeal swab for reverse transcription polymerase chain reaction testing. She had been experiencing cerebrospinal fluid leakage for 1 year after a nasopharyngeal swab performed on an unidentified encephalocele. Antibiotic treatment followed by surgical repair of the encephalocele led to full recovery. Although nasopharyngeal swab tests seem simple, clear instructions are essential for both sample collectors and patients, and any complication, even minor, must be carefully considered. Bacterial meningitis is a serious disease that can cause death or irreversible neurological sequelae. The presence of clear fluid flow after a nasopharyngeal swab should prompt investigation for a meningeal breach. Detecting such a breach may reveal a pre-existing or newly formed malformation, allowing for specialized care to prevent severe complications such as meningitis.

## Introduction

In 2020, at the height of the severe acute respiratory syndrome coronavirus 2 (SARS-CoV-2) pandemic, the World Health Organization reported over 645 million [1] reverse transcription polymerase chain reaction (RT-PCR) tests using nasopharyngeal swabs worldwide. At the peak of the coronavirus disease 2019 (COVID-19) waves in France [2], approximately 4 million tests were conducted weekly. Although the major waves have subsided, the virus continues to circulate, necessitating diagnostic tests to differentiate COVID-19 from other viral respiratory infections such as influenza, respiratory syncytial virus or metapneumovirus. These tests are performed using nasopharyngeal swabs. While this sampling method appears simple and harmless, it carries potential mechanical and infections risks. In France, *Streptococcus pneumoniae* remains the leading cause of bacterial meningitis in adults, affecting approximately one person per 100,000 inhabitants in Metropolitan France in 2022 [3].

In France, pneumococcal vaccination has been mandatory for newborns since 2018. It is also recommended for immunocompromised individuals and adults aged over 65 years [4] [5]. Across all age groups, the incidence of invasive pneumococcal infections decreased from 9.1 to 4.1 cases per 100,000 inhabitants between the pre-vaccination period and 2021 [5], [6].

Risk factors for developing pneumococcal meningitis besides age and immunosuppression are less well known, especially when they are not clinically apparent. Minor trauma during diagnostic procedures, particularly in individuals with certain anatomical anomalies such as encephaloceles (congenital, traumatic or surgical), may facilitate pathogen entry into the meninges. This is especially relevant during nasopharyngeal sampling for respiratory pathogens, including SARS-CoV-2, influenza and others.

We report a case of pneumococcal meningitis occurring after a nasopharyngeal swab complicated by a meningeal breach.

## Clinical case

A 57-year-old woman was admitted to the emergency room in March 2021 for fever, headache and altered consciousness (her initial Glasgow coma scale score was 10/15: 3/3/4) without focal signs. Due to her altered mental state, she was transferred to the intensive care unit at Louis Mourier hospital (Colombes, APHP, France).

Her medical history included hypertension treated with spironolactone, controlled epilepsy and a single dose of pneumococcal vaccine administered in 2016. On physical examination, she exhibited a fever of 39°C, neck stiffness and altered consciousness without signs of focal neurological deficits, purpura or hemodynamic instability.

Laboratory tests showed elevated inflammatory markers: leukocytosis reaching 13 Giga/L (normal values 4–10 Giga/L), polynuclear neutrophils 12 G/L and C-reactive protein at 53 mg/L. Coagulation parameters were normal.

A lumbar puncture revealed cloudy cerebrospinal fluid (CSF). Pneumococcal antigen was detected in the CSF and culture confirmed *S. pneumoniae*.

Brain computed tomography without injection was normal.

A diagnosis of pneumococcal meningitis was established.

Management included resuscitative measures such as orotracheal intubation, antibiotic therapy with cefotaxime and intravenous treatment with dexamethasone.

The patient’s condition improved within 5 days: she became afebrile, regained normal alertness after sedation and her inflammatory markers decreased. She had no neurological sequelae and was transferred from intensive care to internal medicine.

Given the severity of the infection, potential risk factors and predisposing conditions were investigated.

No immunosuppression or relevant medical history were identified. Notably, the patient reported having undergone a nasal RT-PCR test for SARS-CoV-2 1 year earlier, followed by persistent unilateral rhinorrhea, initially attributed to allergic rhinitis.

**Fig. 1:**
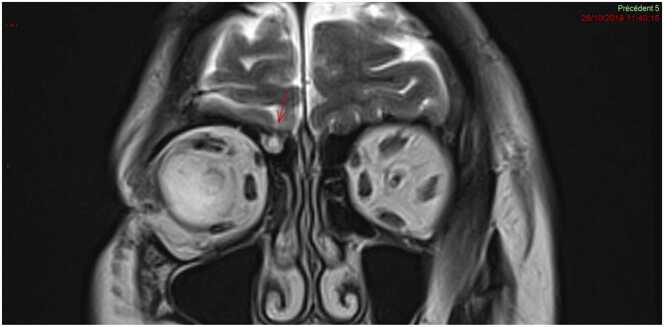
Imaging prior to the CSF leak. Cerebral MRI (T2 sequence) from 2019 in the coronal plane demonstrating a meningocele (arrowhead) situated under the right ethmoid prior to nasopharyngeal testing for COVID-19. COVID-19, coronavirus disease 2019; CSF, cerebrospinal fluid; MRI, magnetic resonance imaging.

**Fig. 2:**
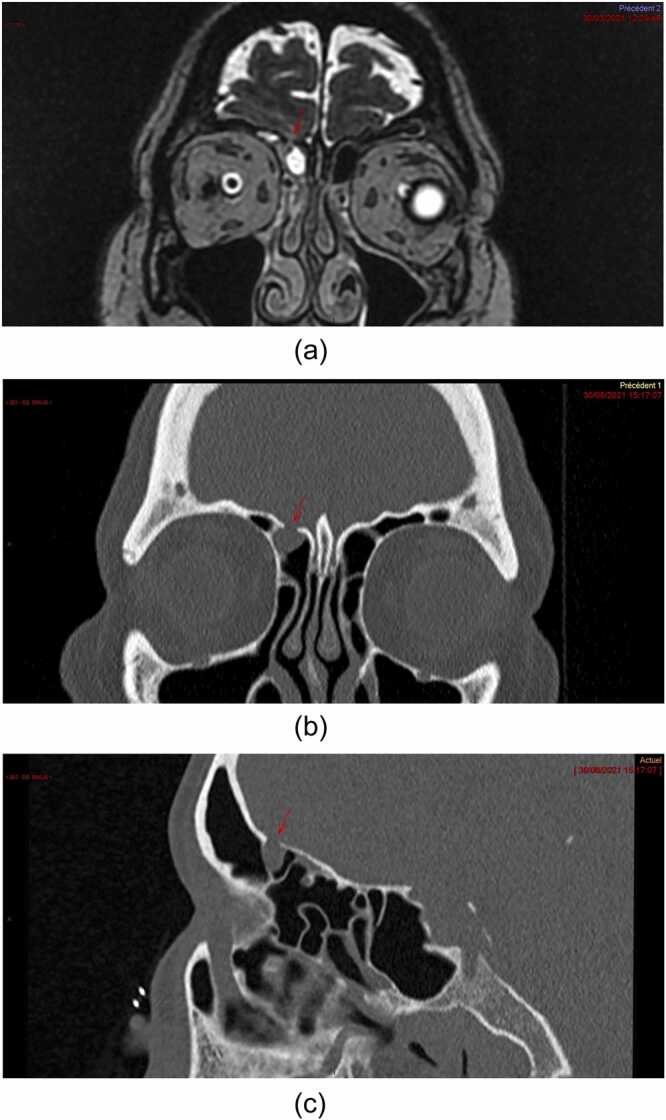
Imaging after the CSF leak. **A.** MRI, coronal view: head MRI in the coronal plane (T2 sequence) demonstrating a meningocele (arrowhead) situated under the right ethmoid. **B.** CT, coronal view: brain CT image in the coronal view demonstrating the right ethmoid breach (arrowhead). **C.** sagittal view: brain CT image in the sagittal view demonstrating the breach and the meningocele (arrowhead). CSF, cerebrospinal fluid; CT, computed tomography; MRI, magnetic resonance imaging.

Re-examination of prior brain magnetic resonance imaging scans performed in 2018 (Fig. 1) revealed a meningocele with a breach in the right ethmoid bone. Biochemical analysis of the rhinorrhea confirmed that it was CSF. We concluded that the breach was caused by the initial sampling, and that the subsequent sampling further weakened the defect (Fig. 2), facilitating pathogen entry and leading to meningitis.

The skull base defect was surgically repaired in September 2021 with no recurrence of CSF leakage.

## Discussion

Bacterial meningitis is a possible serious complication of a nasopharyngeal swab. This sample collection is not necessarily trivial because it can be responsible for severe complications. Some malformations may be contributing factors. More than 12 months of clear rhinorrhea after nasopharyngeal sampling should raise suspicion of a possible neuromeningeal breach. Nasopharyngeal swab RT-PCR is the most widely used test for the diagnosis of COVID-19 and other respiratory pathogens [7]. A Pubmed search conducted on March 5, 2025 using all possible combinations of the search terms “complications”, “adverse events”, “adverse effects” and “nasal swab”, “nasopharyngeal swab”, “oropharyngeal swab” found a review by Kim et al. [8], which revealed several cases of minor trauma responsible for epistaxis and broken swabs requiring removal by ear, nose and throat doctors, one case of a swallowed swab requiring a digestive endoscopy for the removal [9], [10] and cases of CSF leakage after nasopharyngeal sampling for COVID-19 diagnosis, some of which involved previously unknown encephaloceles. A documented case of CSF leakage with meningitis without germ, which was well managed with corticosteroids and antibiotics, has also been described [11]. It should be noted that this patient has previously received antibiotic treatment, which may explain the absence of germs in microbiological samples. Other traumatic cases have been reported in the literature by Vasilica et al. [12] in a patient with intracranial hypertension (IH) of unknown origin with encephalocele mostly repaired surgically and one complicated case of meningitis treated medically without detailed information on germ identification. Clark et al. [13], in a 2021 review, described minor side effects: Epistaxis, nasal discomfort, headache, ear discomfort, rhinorrhea, retained swabs, CSF leak and 1 case of CSF leak complicated by meningitis

Föh et al. [14], described two cases of swab’s tip broken and a left temporomandibular joint dislocation during an oropharyngeal swab.

Hakimi et al. [15], in a retrospective study and literature review, identified 128 cases of minor side effects (fractured nasopharyngeal swab, nasal septum abscess, epistaxis, nasal discomfort, headache, ear discomfort and rhinorrhea) and four cases of CSF leakage including one case complicated by meningitis without germ documented.

Asiri et al. [16] also described, in 2021, a case of meningitis without germ documented 4 months after a nasopharyngeal swab in a patient with known IH 3 years earlier. Holmes et al. [17] reported *Streptococcus salivarius* meningitis complicating nasopharyngeal sampling in a patient with encephalocele.

To our knowledge, apart from this case of meningitis with documented germ presence, no other cases of infectious meningitis with microbiological documentation have been reported in the literature (Table 1). This patient did not have known immunosuppression, in accordance with current pneumococcal vaccination recommendations.

**Table 1:** Characteristics of meningitis after nasopharyngeal swab in the literature.

**Authors/year**	**Patients (age and gender)**	**Pre-existing cerebral malformation**	History of CSF leak	**meningitis?**	**bacteria**	**Treatment**	**Evolution**
Alberola-Amores et al.l 2020	41, F	Encephalocele	Yes 2 months	Yes Diagnosed but complicated meningitis injury waiting for surgery	*Streptococcus salivarius*	appropriate antibiotics	Favorable Restorative surgery
Holmes A. et al.l 2021	54, F	No	Yes 7 months	Yes Unknown prior to meningitis	No	antibiotics	Favorable Restorative surgery
Mohammed Asiri et al.l 2021	36, F	Intracranial hypertension with empty sella turcica in 2018	Yes 4 months	Yes Lesion known 3 years before meningitis	No	Probabilistic antibiotics	FavorableNo surgery
Affo C. et al.l 2025	57, F	Encéphalocele	Yes 12 months	Yes Lesion unknown before meningitis	*Streptococcus pneumoniae*	appropriate antibiotics	Favorable Restorative surgery

Deitmer et al. [7], in their meta-analysis on "Nasal and pharyngeal swab techniques during the COVID-19 pandemic", concluded that clear instructions for performing the nasopharyngeal swab help ensure good quality results and reduce complications more effectively than the qualification of the person collecting the sample. Taking these conclusions into account, and given the seriousness of the complication in our patient, we recommend training for swab collectors and providing clear information on the risks and symptoms suggestive of complications to perform this test safely. We also advise considering all complications, even minor ones, as potential alerts. Our patient experienced a CSF leak during the first sampling, and persistent leakage could have led to the diagnosis of meningocele and its surgical management. This diagnosis might also have prompted an update to our patient's vaccinations.

## Conclusions

Due to the global pandemic caused by SARS-CoV-2 (COVID-19) and seasonal outbreaks of influenza and bronchiolitis, many RT-PCR tests from nasopharyngeal samples are performed. Although the number of COVID-19 cases has diminished, there remains a continuous background of infection, and outbreaks of influenza and bronchiolitis continue to occur and these use the same diagnostic techniques. Indeed, RT-PCR, the gold standard for diagnosing COVID-19 and other respiratory pathogens, appears simple and safe but may be responsible for possible serious side effects. Even minor adverse effects must be considered, and contributing factors should be investigated. Rhinorrhea following a nasopharyngeal swab should prompt investigation for communication with the CSF space and a treatable underlying cause.

## Author contributions

*Drafting of the manuscript*: Comlan Affo. *Critical revision of the manuscript for important intellectual content*: Isabelle Mahé, Comlan Affo, Jeanne Tisseau des Escotais and Antoine Bosquet. Dr. Comlan Affo and Dr. Jeanne Tisseau des Escotais had full access to all of the data in the study and take responsibility for the integrity of the data and the accuracy of the data analysis.

## CRediT authorship contribution statement

**Isabelle Mahé:** Validation, Supervision. **Antoine Bosquet:** Validation, Supervision. **Jeanne Tisseau des Escotais:** Validation, Investigation, Data curation, Conceptualization. **AFFO Comlan:** Writing – original draft, Visualization, Validation, Methodology, Investigation, Funding acquisition, Formal analysis, Data curation, Conceptualization.

## Declaration of Competing Interest

Dr Mahé reports grants from BMS Pfizer and LEO Pharma, personal fees and nonfinancial support from BMS Pfizer, LEO Pharma, Bayer and Sanofi outside the submitted work. Dr Bosquet reports grants from Chugai Pharma France in 2022 outside the submitted work. Drs Affo and Tisseau des Escotais declare no conflicts of interest.
